# Emergency thoracic aortic stent grafting for acute complicated type B aortic dissection after a previous abdominal endovascular aneurysm repair

**DOI:** 10.1186/s40792-015-0096-3

**Published:** 2015-10-07

**Authors:** Ryosuke Yoshiga, Koichi Morisaki, Yutaka Matsubara, Keiji Yoshiya, Kentaro Inoue, Daisuke Matsuda, Yukihiko Aoyagi, Shinichi Tanaka, Jun Okadome, Takuya Matsumoto, Yoshihiko Maehara

**Affiliations:** Department of Surgery and Science, Graduate School of Medical Sciences, Kyushu University Maidashi3-1-1, Higashi-ku, Fukuoka, 812-8582 Fukuoka Japan

**Keywords:** TEVAR, Complicated dissection

## Abstract

We report a case of acute type B aortic dissection with the complication of bowel ischemia and abdominal stent graft compression treated by emergency thoracic aortic stent grafting after endovascular aneurysm repair (EVAR) for abdominal aortic aneurysm (AAA). A 69-year-old male was admitted to our hospital for sudden thoraco-abdominal pain. He had past treatment history of EVAR for AAA half a year ago. A computed tomography (CT) showed acute type B aortic dissection, and conservative treatment was initially performed. Three days after occurrence of aortic dissection, worsened abdominal pain and melena were observed. CT showed that the true lumen and abdominal stent graft was compressed by the false lumen. Emergency thoracic endovascular repair (TEVAR) was performed to close the entry tear. After the operation, the image views and the symptoms were improved. The state was still stable 6 months later. TEVAR for acute type B aortic dissection can become one of the effective treatments.

## Background

Complicated acute type B aortic dissection is a lethal disorder that requires emergency treatment [1, 2]. Recently, the effectiveness of treatment by thoracic stent grafting has been reported [3, 4]. We herein report a case of acute type B aortic dissection complicated by bowel ischemia and abdominal stent graft compression that was treated by emergency thoracic aortic stent grafting after a previous endovascular aneurysm repair (EVAR) for an abdominal aortic aneurysm.

## Case presentation

**Fig. 1:**
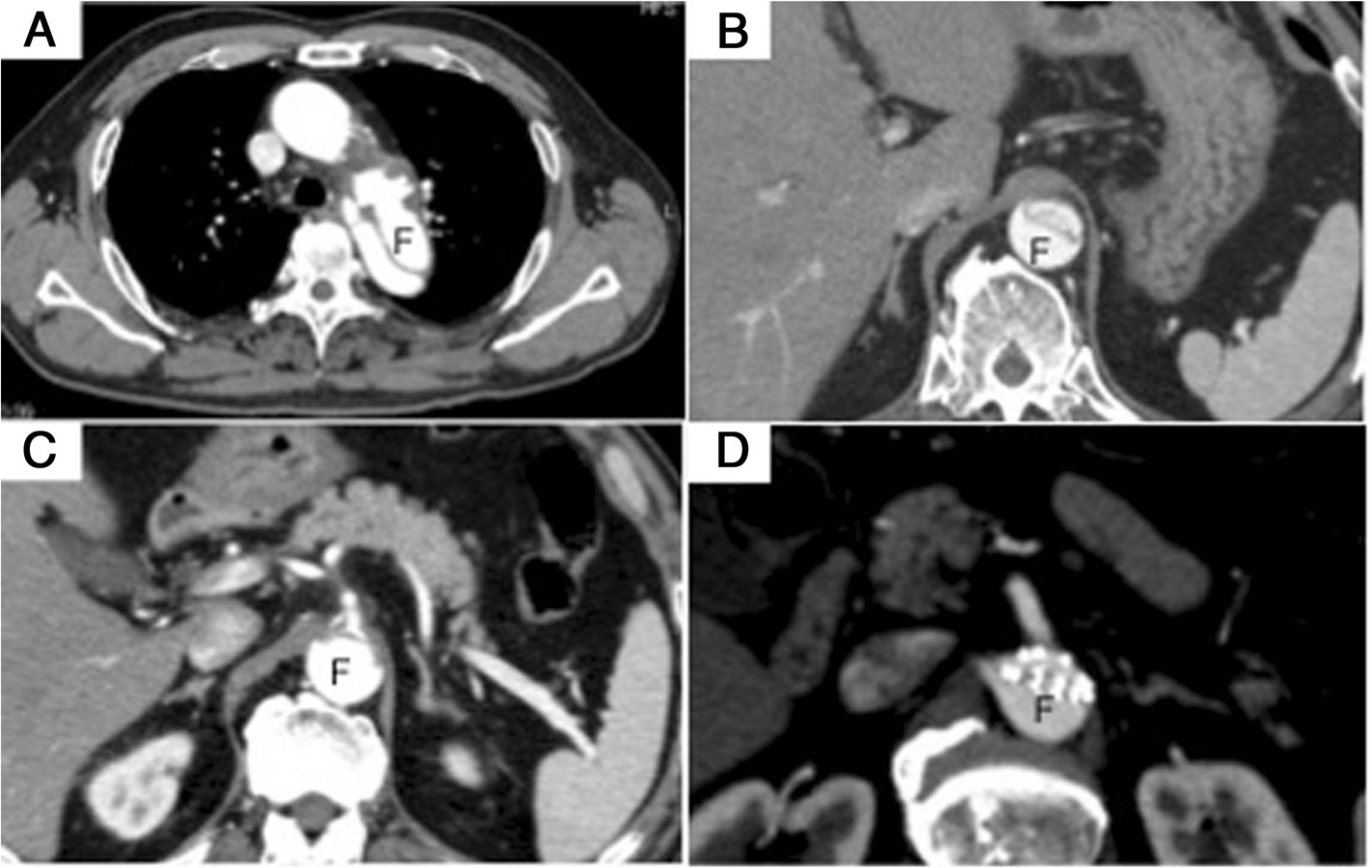
Preoperative CT images of aorta. The true lumen and the abdominal stent graft are compressed by the false lumen. “*F*” indicates false lumen. **a** CT image of thoracic aorta. **b** CT image of abdominal aorta. **c** CT image at the level of the celiac artery. **d** CT image at the level of the deformed previously abdominal stent graft (ENDURANT). Superior mesenteric artery (SMA) is shown with poor contrast effects suggesting SMA occlusion

A 69-year-old male with hypertension and diabetes mellitus complained of sudden thoraco-abdominal pain. He had a treatment history of endovascular aneurysm repair (EVAR) with ENDURANT II bifurcated AAA stent graft systems (Medtronic, Inc., Minneapolis, MN, USA) for an abdominal aortic aneurysm 6 months previously. A computed tomography (CT) scan showed acute type IIIb aortic dissection, which occurred from distal of the left subclavian artery to the renal artery. Conservative treatment was initially performed in another hospital. Three days after the occurrence of aortic dissection, worsened abdominal pain and melena were observed. CT showed that the true lumen was compressed by the false lumen (Fig. 1), especially at the level of the celiac artery (CA) (Fig. 1c). The abdominal stent graft was compressed by the false lumen, and superior mesenteric artery (SMA) was obstructed (Fig. 1d). He was transferred to our hospital immediately. On this admission, the abdominal pain was improved and the pulse of the bilateral femoral arteries was normal. However, ischemia of the left lower leg gradually occurred. Therefore, emergency thoracic endovascular repair (TEVAR) was performed to close the entry tear.

Under general anesthesia, Gore TAG (TGU373715J, W.L. Gore & Associates, Inc., DE, USA) was deployed just distal of the left common carotid artery and left subclavian artery was embolized with an AMPLATZER Vascular Plug (9-PLUG-016, ST. Jude Medical, MN, USA). The left leg of the abdominal stent graft, which was implanted previously, was occluded by thrombus due to compression by the false lumen. Additional stent graft (ENDURANT II, ETLW1624C124EJ, Medtronic, MN, USA) implantation into the left leg of the abdominal stent graft and deployment of E-LUMINEXX (ZVM14060, C.R. Bard, NJ, USA) and Epic (39200-10607, Boston Scientific, MA, USA) were performed.

**Fig. 2:**
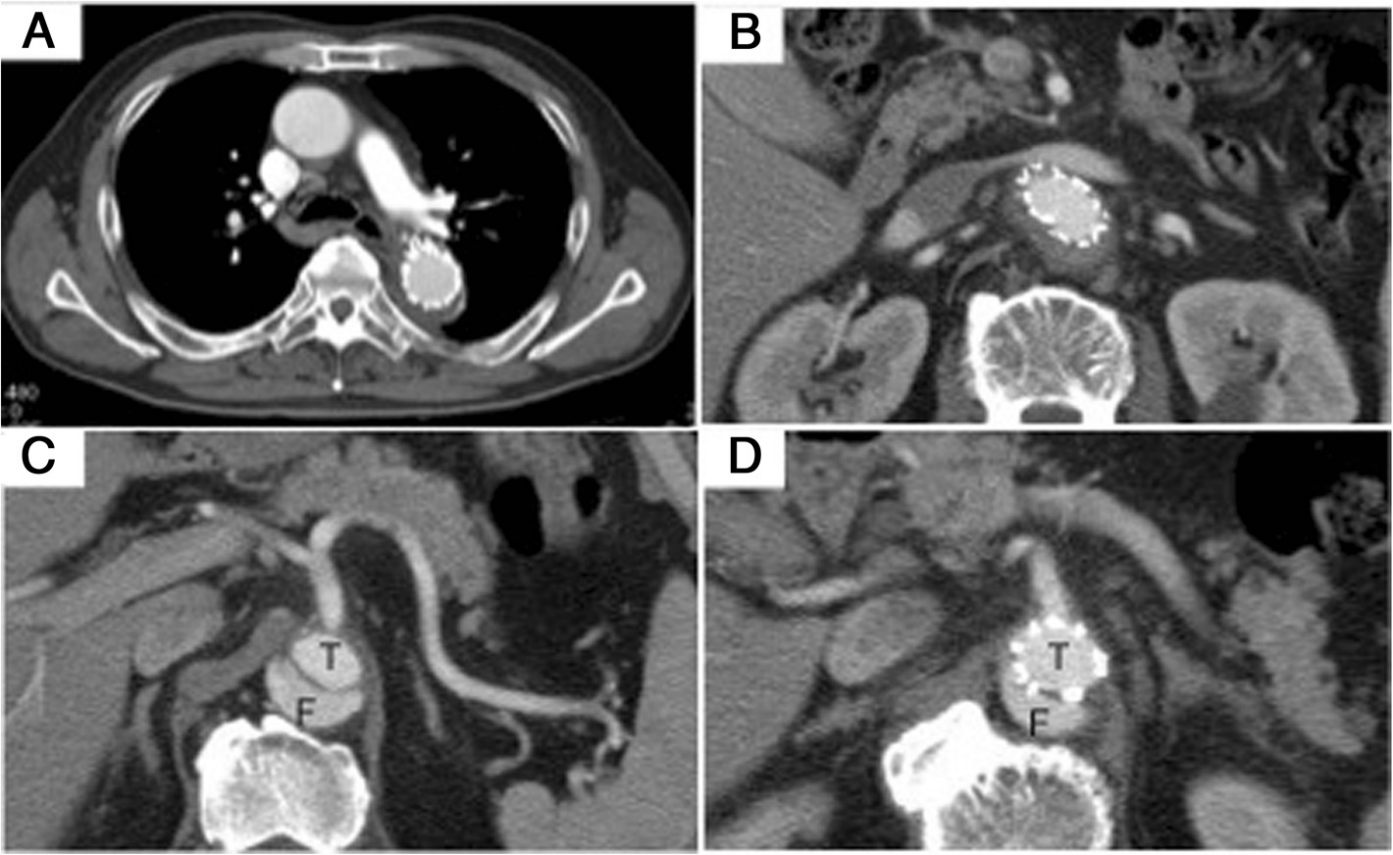
Postoperative CT images of aorta at 6 months later. Compressions of the true lumen and the abdominal stent graft are improved. The false lumen is partially thrombosed without any dilatation of the false lumen. “*F*” indicates false lumen. “*T*” indicates true lumen. **a** CT image of thoracic aorta. **b** CT image of abdominal aorta. **c** CT image at the level of the celiac artery. **d** CT image at the level of the expanded previously abdominal stent graft (ENDURANT). Superior mesenteric artery (SMA) is shown with good contrast effects suggesting improved the blood flow of SMA

The abdominal pain and ischemia of the left leg were improved following the emergency operation. Postoperative CT at 6 months later showed the false lumen was partially thrombosed without any dilatation of the false lumen (Fig. 2).

### Discussion

Complicated type B aortic dissection is a lethal disorder, which requires immediate endovascular or surgical treatment. The present case had acute type B aortic dissection with ischemia of the bowel and lower extremity after previous EVAR, and we treated it by thoracic stent graft to close entry and false lumen.

We experienced very rare case of complicated type B aortic dissection with the deformation of abdominal stent graft. It seemed that radial force of previous abdominal stent graft was lower than the pressure of false lumen. The true lumen was expanded and the blood flow of CA and SMA were improved by entry closure with thoracic stent graft (Gore TAG). However, the blood flow of lower limb was not improved, and deformed previous abdominal stent graft was remained. Thus, additional implantations of abdominal stent graft and bare metal stents were consequently needed. Considering these situations, the pressure of false lumen would be too high, and bare metal stent may be necessary in case of persistent malperfusion after entry closure.

Recently, endovascular treatment has become more common, and several reports have shown the efficacy of TEVAR according to preferred operative mortality compared to open repair [2, 3]. Fattori et al. demonstrated that the operative mortality and complication were 33.9 and 40 % in open surgery compared to 10.6 and 20 % in TEVAR [4], respectively. Another report showed that the 30-day mortality rate was 17.5 % in open repair and 10.2 % in TEVAR [5].

In the present case, CA, SMA, and lower leg malperfusion occurred due to compression of the true lumen by the false lumen. The patient had symptoms due to CA, SMA, and leg obstruction that needed prompt revascularization. Entry tear was detected distal of the left subclavian artery, so we thought stent graft could close entry to reduce false lumen. Several reports showed the efficacy of entry closure by a stent graft in cases of complicated type B aortic dissection [4, 6]. Therefore, we thought the stent graft was appropriate treatment compared to open repair.

After TEVAR, the blood flow of CA and SMA was improved; however, ischemia of the left leg remained with compressed previous abdominal stent graft. Implantation of the left leg with the abdominal stent graft was unable to improve the blood flow of the left lower limb. To increase the radial force, self-expandable nitinol stents (E-LUMINEXX and Epic) were implanted into the left leg, which successfully improved the blood flow of the left lower limb. Considering compression of the abdominal stent graft by dissection, there may be cases in which entry closure is insufficient treatment for type B dissection with CA, SMA, and leg obstruction. On the other hand, physicians should be attentive to the enlargement of dissection by implantation of stents.

The patient’s symptoms were improved after the operation, and postoperative CT showed good CA, SMA, and legs blood flow; however, the false lumen was patent without increase. Entry closure by a stent graft can be effective; however, the preventive benefit of an aneurysm rupture remains unclear. After treatment of TEVAR, approximately 25 % of the patients require reintervention due to endoleaks and increasing false lumens in size [7–9]. Furthermore, a ruptured false lumen was found to be an independent risk factor for the long-term survival [10]. Therefore, patients with a patent false lumen should be followed up carefully.

The percentage of thrombosed false lumen is approximately 31–79 % of previous reports [6, 11, 12]. Regarding the thrombus formation of a false lumen after entry closure, within 6 months from the onset of dissection, the false lumen reduced in size [13, 14]. Therefore, the preferred treatment seems to be entry closure by a stent graft within 6 months compared to after 6 months from the aortic dissection. However, the risk factor for a patent false lumen in acute complicated type B aortic dissection remains unclear.

Entry closure by TEVAR for complicated acute type B aortic dissection is thus considered to be effective; however, several problems can occur in cases which required reintervention. Further studies must be performed to determine the long-term outcomes and risk factors for reintervention.

## Conclusions

We experienced very rare case of complicated type B aortic dissection with the deformation of abdominal stent graft. Entry closure with thoracic stent grafting was an effective treatment for acute type B aortic dissection with celiac artery, superior mesenteric artery, and lower leg malperfusion. Careful follow-up is needed in the case of a patent false lumen.

## Consent

Written informed consent was obtained from the patient for publication of this case report and any accompanying images. A copy of the written consent is available for review by the Editor-in-Chief of this journal.

## Abbreviations

AAA: abdominal aortic aneurysm
CA: celiac artery
CT: computed tomography
EVAR: endovascular aneurysm repair
SMA: superior mesenteric artery
TEVAR: thoracic endovascular repair
